# The effect of seabird presence and seasonality on ground‐active spider communities across temperate islands

**DOI:** 10.1002/ece3.9570

**Published:** 2022-12-03

**Authors:** Penelope Pascoe, Melissa Houghton, Holly P. Jones, Christine Weldrick, Rowan Trebilco, Justine Shaw

**Affiliations:** ^1^ Institute for Marine and Antarctic Studies University of Tasmania Battery Point Tasmania Australia; ^2^ Department of Natural Resources and Environment Tasmania New Town New South Wales Australia; ^3^ Biological Sciences and Institute for the Study of the Environment, Sustainability, and Energy Northern Illinois University DeKalb Illinois USA; ^4^ Australian Antarctic Program Partnership University of Tasmania Hobart Tasmania Australia; ^5^ CSIRO Oceans & Atmosphere Hobart Tasmania Australia; ^6^ School of Biology and Environmental Sciences Queensland University of Technology Brisbane Queensland Australia

**Keywords:** Araneae, biodiversity assessment, community composition, invertebrates, island biogeography, seabirds

## Abstract

Seabirds influence island ecosystems through nutrient additions and physical disturbance. These influences can have opposing effects on an island's invertebrate predator populations. Spiders (order: Araneae) are an important predator in many terrestrial island ecosystems, yet little is known about how seabird presence influences spider communities at the intraisland scale, or how they respond to seasonality in seabird colony attendance.We investigated the effects of seabird presence and seasonality on ground‐active spider community structure (activity‐density, family‐level richness, age class, and sex structure) and composition at the family‐level across five short‐tailed shearwater breeding islands around south‐eastern Tasmania, Australia. Using 75 pitfall traps (15 per island), spiders were collected inside, near, and outside seabird colonies on each island, at five different stages of the short‐tailed shearwater breeding cycle over a year. Pitfall traps were deployed for a total of 2674 days, capturing 1592 spiders from 26 families with Linyphiidae and Lycosidae the most common. Spider activity‐density was generally greater inside than outside seabird colonies, while family‐level richness was generally higher outside seabird colonies. For these islands, seabird breeding stage did not affect activity‐densities, but there were some seasonal changes in age class and sex structures with more adult males captured during winter. Our results provide some of the first insights into the spatial and temporal influences seabirds have on spider communities. We also provide some of the first records of spider family occurrences for south‐eastern Tasmanian islands, which will provide an important baseline for assessing future change.

## Graphical abstract

We provide some of the first insights into the spatial and temporal influences seabirds have on spider communities. We also provide some of the first records of spider family occurrences for south‐eastern Tasmanian islands, which will provide an important baseline for assessing future change.

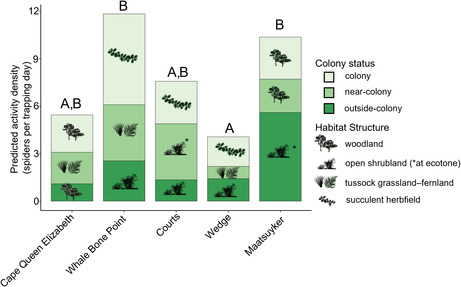

## 1 INTRODUCTION

Seabird islands are recognized as distinctive ecosystems with global significance, driven by the often large numbers of seabirds breeding on them (Mulder, Anderson, et al., 2011). Foraging at sea and returning to islands to breed, seabirds transport marine‐derived nutrients to islands via guano deposition, carrion, feathers, and regurgitate (Smith et al., 2011). These marine‐subsidies influence nutrient dynamics throughout terrestrial island food webs, often increasing primary productivity (Polis et al., 1997; Stapp et al., 1999) and consumer abundances (Kolb et al., 2011; Polis & Hurd, 1995; Sanchez‐Pinero & Polis, 2000; Towns et al., 2009). Seabirds also influence island ecosystems through physical disturbance (Smith et al., 2011). Burrowing, nesting, and trampling can reduce vegetation richness and habitat complexity (Ellis et al., 2011; Kolb et al., 2010), which can reduce invertebrate herbivore activity‐density and diversity (Toft & Schoener, 1983; Tsai et al., 2006; Vanbergen et al., 2017). Soil and litter moisture (Fukami et al., 2006) and soil chemistry (Mulder, Jones, et al., 2011) can also be altered, making conditions unfavorable for some invertebrate taxa (Kolb et al., 2011). Nutrient additions and physical disturbance by seabirds have flow on effects to predatory invertebrates (Kolb et al., 2011; Polis & Hurd, 1995). While seabird‐mediated nutrient subsidization may increase productivity and consumer abundance, the negative effects of seabirds on soil chemistry and habitat complexity can make the responses of predatory taxa, such as spiders, less predictable (Kolb et al., 2010, 2011).

Spiders (order Araneae) are an essential component of terrestrial ecosystems. Abundant and taxonomically rich, all are predatory yet display a diverse array of foraging strategies and fill a large variety of niches (Branco & Cardoso, 2020; Marc et al., 1999; New, 1999). Spiders provide an important food source for higher trophic levels, and are important predators in most terrestrial ecosystems, particularly on islands where native mammals and reptiles can be absent (Nyffeler & Birkhofer, 2017; Toft & Schoener, 1983). To better understand how terrestrial island ecosystems function, we need to understand how seabirds influence spider communities. While several studies have compared spider communities on islands with and without seabirds, the responses of spider abundance, richness, and community composition have been variable (Kolb et al., 2010; Markwell & Daugherty, 2002; Polis & Hurd, 1996, 1995; Towns et al., 2009), and less is known about how seabirds influence spider communities at the intraisland scale (but see Polis & Hurd, 1996).

The influence of seabirds on an island is not homogenous. Most seabirds are colonial nesters (Danchin & Wagner, 1997), meaning some regions of an island may have dense seabird colonies, while other regions are seabird free. Understanding localized effects of seabird presence on invertebrate communities is important for understanding the terrestrial processes on offshore islands and the island‐wide conservation implications of seabird population fluctuations.

In addition to being spatially heterogeneous, seabirds are also seasonal. During the breeding season, adult seabirds spend time at their breeding island incubating, guarding, and provisioning eggs and chicks. Once chicks have fledged and adults have departed for winter foraging grounds, a colony may then remain empty for many months (Skira, 1991). This leads to large fluctuations in nutrient deposition and physical disturbance. How spider communities, particularly ground‐active spiders, those which are most impacted by the physical disturbances, respond to seasonality in seabird colony attendance is largely unknown.

Despite being among the most common animals in terrestrial habitats and having crucial ecological roles, spiders are underrepresented in conservation and monitoring programs and ecological research (Cardoso, 2012; Cardoso et al., 2011; Milano et al., 2021). Where monitoring has occurred, such as in Great Britain, substantial declines in common species have been detected (Harvey et al., 2017). Spiders are threatened by many anthropogenic factors ranging from agriculture to climate change and pollution (Branco & Cardoso, 2020). Insufficient taxonomic knowledge or taxonomic specialists to identify spiders is common for many regions. This further impedes the inclusion of spiders in ecological monitoring and conservation projects (Cardoso et al., 2011; Ward & Lariviere, 2004).

To overcome this taxonomic impediment, higher taxa can be used as surrogates for species‐level identification (Cardoso et al., 2004; Driessen & Kirkpatrick, 2019; Gaston & Williams, 1993). The level of taxonomic identification made can influence the results of studies using this method. The use of higher taxa is better able to distinguish between communities when levels of disturbance or environmental gradients are stronger, and when the ratio of higher taxa levels to the number of individual species is higher (Bevilacqua et al., 2012). A review of taxonomic surrogacy across 280 invertebrate, algae, and plant case studies found that generally, surrogacy up to the level of family was able to detect changes in communities disturbed naturally or by humans, however for subtle levels of disturbance, finer levels of taxonomic resolution may be required (Bevilacqua et al., 2012). For some regions, sparsity of taxonomic information may be the limiting factor for choosing an identification resolution. Additionally, as higher taxa groupings may contain species occupying various niches, individual species responses to different conditions may be lost by only considering the higher taxonomic level (Ward & Lariviere, 2004). Consequentially, the interpretation of results should always be done with caution.

Located at the south‐eastern tip of Australia, Tasmania is a cool temperate island state (6.8 million ha) surrounded by some 330 offshore islands (>1 ha) (Bryant & Harris, 2021). These offshore islands have high conservation values. They are home to over a third of the state's terrestrial threatened fauna species, support large numbers of breeding seabirds, and provide critical habitat for many species, including several endemics (Brothers et al., 2001; Bryant & Harris, 2021). Little is known about the islands' spider communities and ecology. Taxonomic information on Tasmanian spiders is sparse. Many species remain undescribed (Driessen & Kirkpatrick, 2019) and very few published resources exist for spider taxonomy, with the available references out of date (e.g., Simon, 1903), or covering a limited or incomplete range of taxa (e.g., Douglas, 2008; Hickmas, 1967). Published studies investigating Tasmanian spiders are also limited, focusing only on specific regions (e.g., Bryant & Shaw, 2006; Churchill & Arthur, 1999) or species of interest (e.g., Doran et al., 1999). Tasmania provides a classic example of the taxonomic impediment preventing spiders being included in monitoring studies, particularly on Tasmania's islands where available information is even more limited (Bryant & Harris, 2021). Despite this, obtaining community structure and composition data is essential for informing management and providing baseline data from which to assess future changes.

In this study, we aim to provide the first insights into spider communities on five island locations around south‐eastern Tasmania. Given the current taxonomic limitations we use family‐level taxonomic surrogacy to investigate the effects of seabird colony presence and seasonality in colony attendance on spider communities and how this varies between islands. Specifically, we aim to:

1. Provide a record of ground‐active, family‐level spider communities on under‐studied Tasmanian islands.
2. Investigate the effects of seabird presence, season and habitat structure on ground‐active spider activity‐density, family‐level richness, sex and age class structure and overall community composition at the family‐level, and if this varies between islands.

## 2 METHODS

### 2.1 Study islands

**FIGURE 1:**
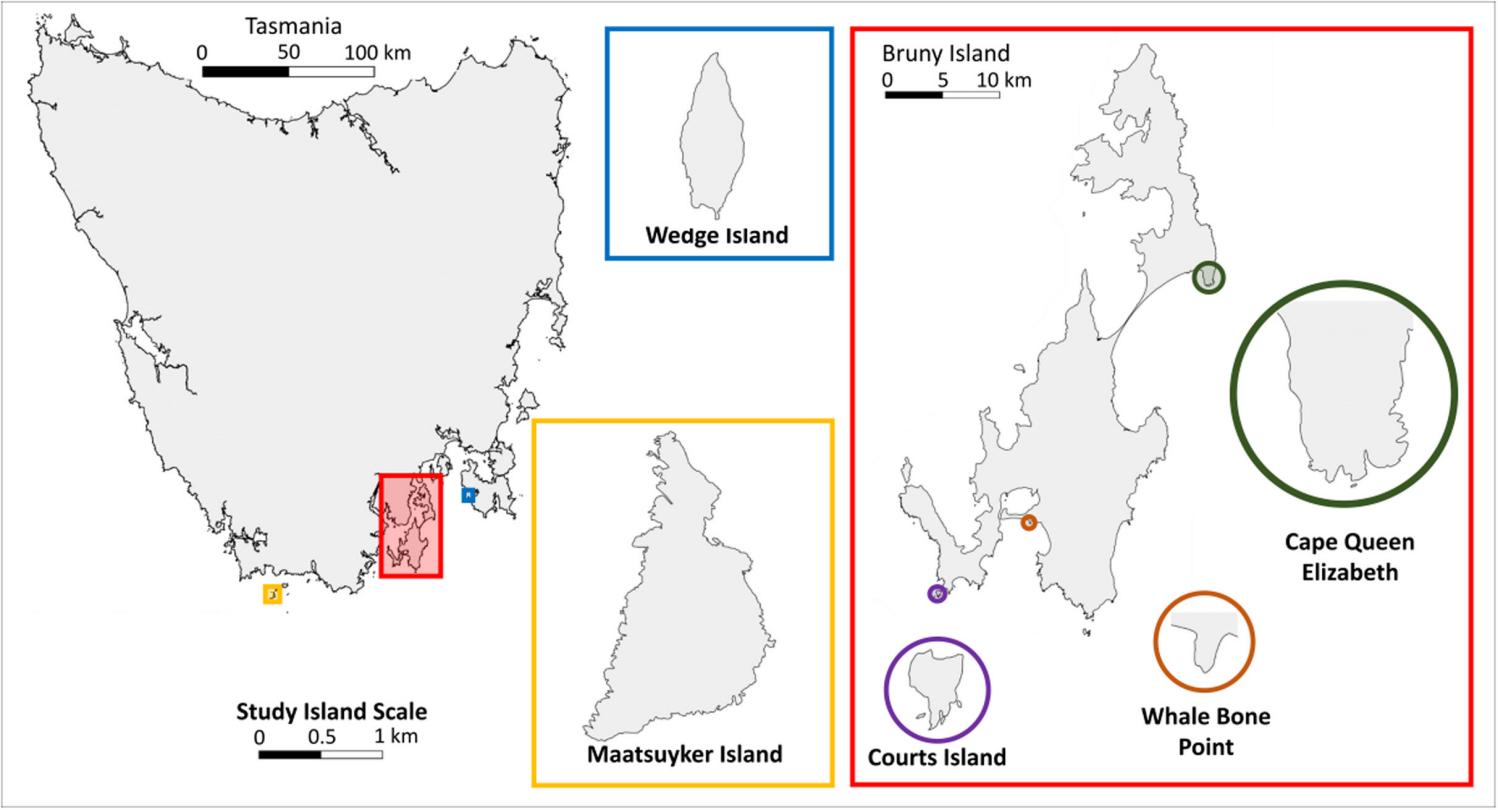
Locations of study islands (in bold) around south‐eastern Tasmania, Australia. Study islands are shown at the same scale.

We sampled ground‐active spiders from five island locations around the south‐east coast of Tasmania, Australia. These included Maatsuyker Island (186 ha) situated 10 km from the Tasmanian mainland, two near‐shore islands (separated by <1 km from the nearest large land mass) Wedge Island (43 ha) and Courts Island (16 ha), and two peninsular sites situated at either end of the large Bruny island (36,200 ha); Cape Queen Elizabeth (north Bruny Island) and Whale Bone Point (south Bruny Island) (all sites hereafter referred to as ‘islands’) (Figure 1, Table 1). The islands span a latitudinal range of just 43.14°S to 43.65°S. Annual mean temperatures range from 13.9°C on Maatsuyker Island to 17.2°C on Wedge Island, and annual mean rainfall ranges from 781.7 mm on Wedge Island to 1223.4 mm on Maatsuyker Island. We assessed the currently available records for spiders on each island (Natural Values Atlas www.naturalvaluesatlas.tas.gov.au, accessed 9 March 2022): five species are recorded for Maatsuyker Island, 28 species for Bruny Island, and no spider species have previously been recorded for Wedge or Courts islands.

**TABLE 1:** (A) Characteristics of island study site. Estimates of the numbers of burrows on each island are sourced from Skira et al. (1996). At each island, spiders were collected at colony, near‐colony, and outside‐colony locations. The dominant habitat structure at each location is indicated with habitat structure class classifications defined in part (B).

(A) Island characteristics						
Island/Peninsula	Area (ha)	Distance from larger landmass	Estimated burrows (range)^a^	Habitat structure (see part B)		
				Colony	Near‐colony	Outside‐colony
Cape Queen Elizabeth (Bruny Island)	~120 (36,200)	Peninsula of Bruny Island	43,475 (26,825–60,125)	Succulent herbfield	Tussock grassland–fernland	Woodland
Courts Island	15.83	<100 m	34,000 (29,200–38,800)	Succulent herbfield	Open shrubland	Open shrubland
Maatsuyker Island	186.15	10 km	800,000 (750,000 –850,000)	Woodland	Woodland	Open shrubland
Wedge Island	42.58	< 1 km	11,400 (7800–15,400)	Succulent herbfield	Tussock grassland–fernland	Open shrubland
Whale Bone Point (Bruny Island)	~6 (36,200)	Peninsula of Bruny Island	9180 (7830–10,530)	Succulent herbfield	Tussock grassland–fernland	Open shrubland
**(B) Habitat structure classes** b						
**Habitat structure**	**Dominant species**			**Height**	**Structural elements**	
Woodland	*Eucalyptus tenuiramis* (Cape Queen Elizabeth) or *Leptospermum scoparium* (Maatsuyker Island)			>3 m	Trees greater than head height forming a canopy with sparse understory	
Open shrubland	*Olearia stellulata, O. ramulosa, Banksia marginata, Ozothamnus reticulatus, Acacia longifolia* and/or *Westringia rigida*			<2 m	Woody scrub below head height	
Tussock grassland–fernland	*Lomandra longifolia* and *Pteridium esculentum*			1–2 m	Tussock and fern dominated with an infrequent and sparse shrub overstorey	
Succulent herbfield	*Tetragonia implexicoma, Rhagodia candolleana,* and/or *Carpobrotus rossii*			5–15 cm	Thick herbaceous mat interlaced with bare ground	

*Note*: Habitat structure classifications are based on the broad plant community classes defined by Walsh et al. (1997) and records made of the dominant species at each site.

^a^ Skira et al. (1996)

^b^ Based on Walsh et al. (1997).

A prominent feature of each island are their burrowing seabird colonies, dominated by short‐tailed shearwaters (*Ardenna tenuirostris*). Colony sizes range from an estimated 7830–10,530 breeding pairs at Whale Bone Point to 750,000–850,000 on Maatsuyker Island (Skira et al., 1996), although updated and more accurate colony size assessments are required. The islands are diverse in their native and nonnative mammal assemblages: Maatsuyker Island has remained invasive mammal free and is home to just one native mammal, the swamp antechinus (*Antechinus minimus*); Wedge Island underwent feral cat (*Felis catus*) eradication in 2004 and is home to the native rodent, rakali (*Hydromys chrysogaster*); and Whale Bone Point, Cape Queen Elizabeth, and Courts Island have many native and nonnative mammalian species present, including cats, rodents, and marsupials (Natural Values Atlas www.naturalvaluesatlas.tas.gov.au, accessed 9 March 2022).

### 2.2 Study design

On each island we established three sampling locations: one situated in a short‐tailed shearwater colony (*colony*), one outside the colony within 20 m of the colony edge (*near‐colony*), and one at more than 70 m from the nearest burrow (*outside‐colony*). Hereafter these are referred collectively to as “locations.” We classified each location into one of four broad habitat classes based both on the dominant plant species present and the overall structural elements of the site, using the classifications made by Walsh et al. (1997) as a guide (Table 1).

Five invertebrate pitfall traps were deployed at each location (*n* = 15 per island). Traps were set at approximately 5 m intervals and consisted of a 7 cm diameter piece of polypipe inserted flush with the ground. A 200 ml plastic beaker was inserted flush with the polypipe and filled with 1 cm propylene glycol. We left traps set for 4–10 days depending on island accessibility (mean = 8.22 days) before collection. The short duration was chosen to minimize the chance of traps flooding from rain, and prevent the degradation of samples which were also being used for stable isotope analysis (see Pascoe et al., 2022). As further protection from flooding, traps were also placed under vegetation where possible to provide shelter from rain. The observed water levels in traps at collection indicated that trap flooding was rarely and issue. Pitfall trap contents were passed through 20 denier polyganza mesh to remove the propylene glycol and then covered with 70% ethanol for preservation. We repeated this process five times over a year, giving a total of 2674 trapping days (15 traps on five islands deployed five times for 4–10 days each deployment). Each sampling event was timed to coincide with a different season and stage of the short‐tailed shearwater breeding cycle: 1. Autumn, March 2020—prefledging; 2. Winter, June/July 2020—start of vacant colony period; 3. Spring, September 2020—end of vacant colony period; 4. Summer, December 2020—incubation and 5. Autumn, March 2021—prefledging.

### 2.3 Sample analysis

We sorted the contents of each pitfall trap under a Zeiss Stemi DV4 Stereo Microscope to extract all of the spiders. These were then identified and sorted by taxon, age class, and sex. Given the paucity of research undertaken on spider taxonomy in Tasmania, we used family‐level taxonomic surrogacy. This resolution was selected as the absence of previous spider studies on these islands means identification below this resolution is near impossible with the currently available resources. Additionally, as seabirds cause significant levels of disturbance (Smith et al., 2011), family‐level surrogacy should be suitable for detecting differences between sites and time periods (Bevilacqua et al., 2012; Driessen & Kirkpatrick, 2019). We assigned spiders to two age class categories: adult (individuals with identifiable genital structures), and immature (small individuals with no recognizable or very underdeveloped genital structures) (Supplementary S1). For immature spiders, sex, and sometimes family, could not be determined and they were classed as “unknown immatures.”

### 2.4 Statistical analysis

All analyses were performed in R studio (RStudio Team 2018) using R version 4.0.1 (R Core Team, 2020). Code and raw data used are available at Pascoe (2022). Means are presented as ± one standard deviation. For each sampling event (*n* = 5), location (*n* = 3 per island) served as the unit of replication, with the spiders caught from the five pitfall traps set at each location summed to provide a single value for each sampling event. To account for variability in the number of days pitfall traps were deployed between islands and sampling events, we standardized pitfall trap catches by dividing the summed catch at each location (the total spiders caught across the 5 traps) by the number of days the traps were open on that island for that sampling event. All further analysis was conducted on these standardized activity‐density numbers.

Statistical analysis was conducted in two parts. (1) We investigated differences in spider family‐level community structure between islands, locations, habitat classes, and sampling events by comparing which of these variables were the most significant drivers of activity‐density, family‐level richness and age and sex class structure. (2) We then compared the overall similarity of family‐level community composition between each variable using Principal Component Analysis (PCA). Detailed methods for each analysis step are outlined below.

#### 2.4.1 Family‐level community structure

For each island, colony status, habitat structure and sampling event, we calculated spider activity‐density, family richness, and age class and sex structure. Activity‐density was calculated based on standardized spider numbers and included unidentified and damaged spiders. We calculated family‐level richness excluding unidentified and damaged spiders. We then used rarefaction analysis to adjust these scores to account for uneven spider numbers collected, using the “rarefy” function (R package “vegan,” Oksanen et al., 2022). The age class structure was calculated as the proportion of adults to immatures, excluding damaged individuals which could not be assigned to an age class. Sex structure was calculated as the proportion of adult males to adult females, excluding damaged individuals which could not be assigned to a sex.

For spider activity‐density and rarefied family richness, we investigated the effect of island, colony status, habitat structure, and sampling event using linear effects models (R package “stats,” R Core Team, 2020) and linear mixed‐effects models (R package “lme4,” R Core Team, 2020). The differences in spider activity‐densities between islands were first investigated using a linear model. To account for differences between islands, island was then included as a random effect to investigate the effects of colony status, habitat structure, and sampling event on the response variables of spider activity‐density, rarefied family richness, and age class and sex structure, each calculated using standardized spider numbers. Chi‐squared tests using the “drop1” function (R package “stats,” R Core Team, 2020) was used to assess the importance of each predictor variable, with *p*‐values <.05 considered significant. Differences between levels of each predictor variable were assessed using Tukey's posthoc tests (R package “multcomp,” Hothorn et al., 2008).

#### 2.4.2 Family‐level community composition

We used PCA to explore differences in the family community compositions between islands, sampling events, and colony status locations with the unconstrained “rda” function (R package “vegan,” Oksanen et al., 2022). A Hellinger distance matrix was used to allow for nonlinear responses. We plotted results on two principal component axes with separate plots for the groupings of island, sampling event, and colony status, with ellipses representing one standard deviation from the centroid of each group using function “ordiellipse” (R package “vegan,” Oksanen et al., 2022). For completeness, we also conducted non‐metric multi‐dimensional scaling (NMDS) using function “metaMDS” (R package “vegan,” Oksanen et al., 2022) (Supplementary S2). The two methods produced qualitatively similar plots so we only include the PCA results in the main text. We used permutational multivariate analysis of variance (PERMANOVA) using distance matrices to identify if there was a significant difference (*p* < .05) in community family composition between islands, sampling events or colony status locations using the “adonis2” function (R package “vegan,” Oksanen et al., 2022). A Hellinger distance matrix was used with the condition “by margin,” and the model run to 10,000 permutations. Where a significant difference was detected, we preformed two‐way comparisons between each level of each variable using the “adonis. pair” function (R package pairwiseAdonis, Arbizu, 2017). Again, a Hellinger distance matrix was used, and each model run to 10,000 permutations, with a conservative Bonferroni *p*‐value adjustment applied to control for false discovery of differences between groups through compounding Type I error across the multiple tests.

## 3 RESULTS

**FIGURE 2:**
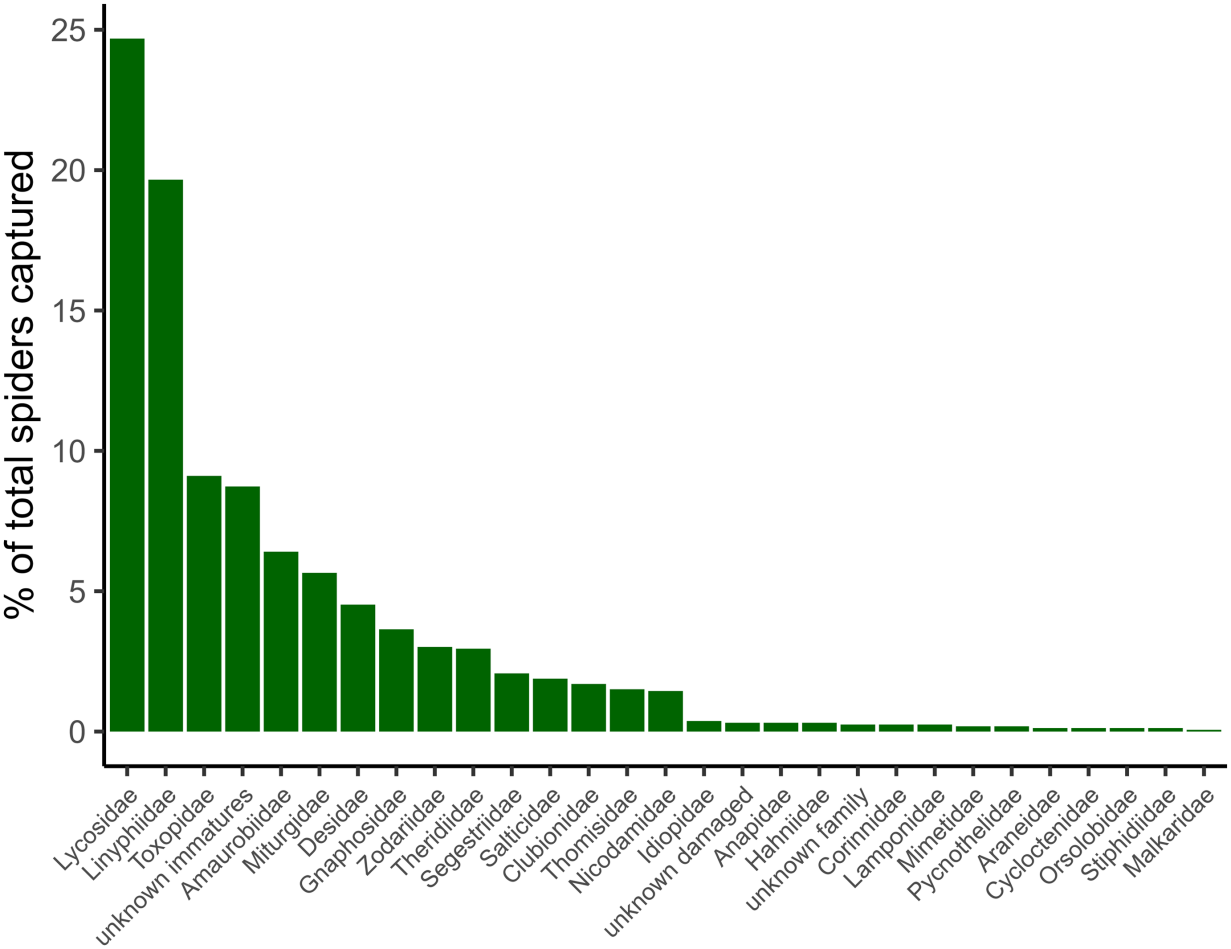
Percentage of the total study‐wide catch comprised by each spider family.

A total of 1592 spiders were collected over 3075 trap days across the five islands, giving a catch rate of 0.52 spiders per trap per day. Of these, 1075 were adult and 490 were immature. Due to specimen damage, 27 individuals could not be assigned to an age class. Of the adults, 58.6% were male and 41.4% female. 90.7% of the spiders (99.6% of adults) were identified to family‐level. Excluding unidentified individuals, 26 families were found across the islands, with Lycosidae and Linyphiidae the most abundant and widespread, representing 24.7% and 19.7% of all spiders, respectively (Figure 2). Linyphiidae were also found across all five islands and all locations within an island, and Lycosidae were found at all locations except for at the *colony* and *outside*‐*colony* locations on Wedge Island (Supplementary S3).

**FIGURE 3:**
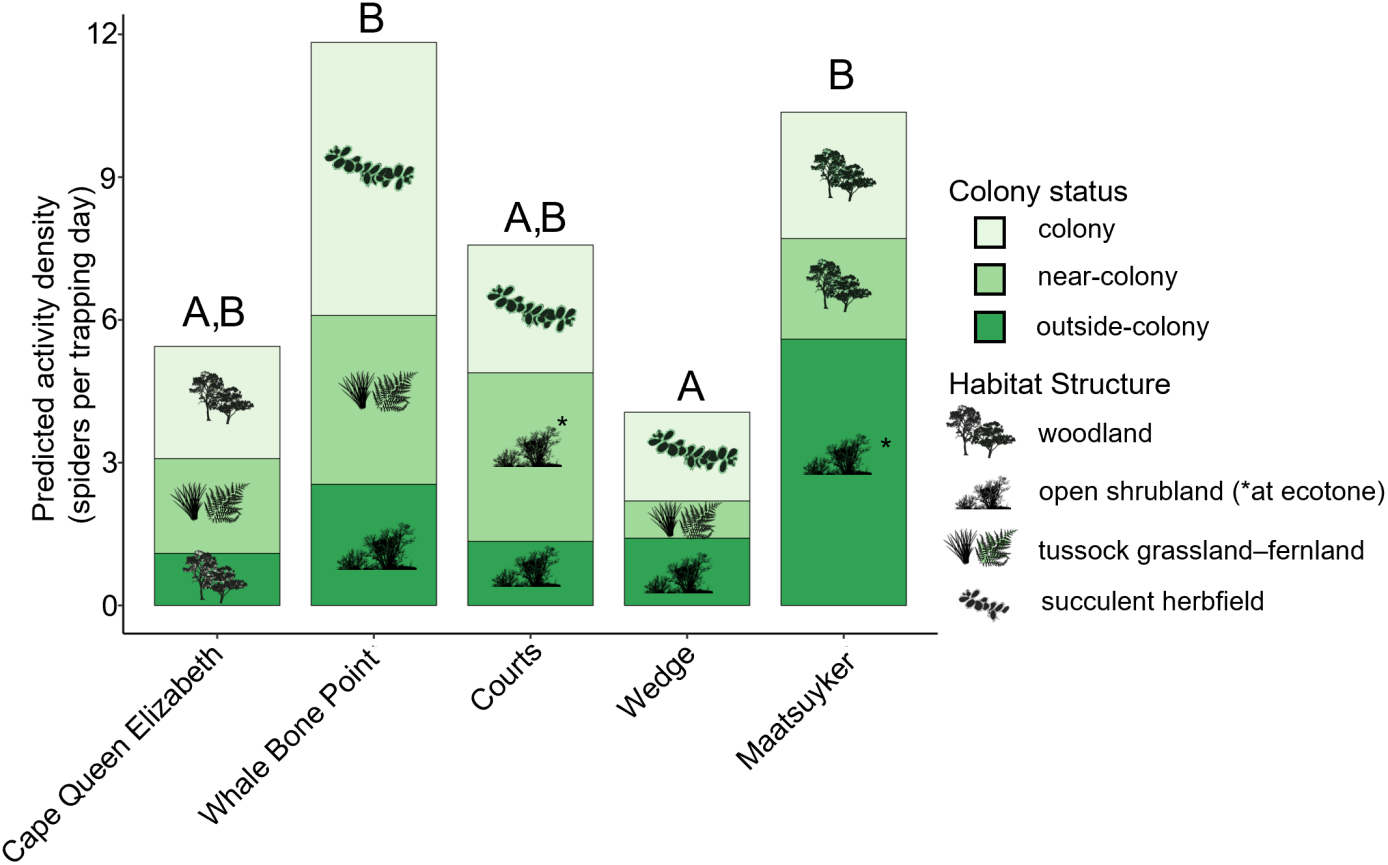
Standardized spider activity‐density (number of spiders captured in a trapping location/trapping days) predicted from the best preforming model: spider activity‐density ~ island*colony status + (1|island). Habitat structure and colony status were highly correlated (Sakoda's adjusted Pearson's Coefficient = 0.90) so the former was not included in the model selection process but is shown here for visual reference. Different letters indicate activity‐densities between those islands were significantly different (*p* < .05).

Colony status and habitat structure were highly correlated (Sakoda's adjusted Pearson's Coefficient = 0.90). Across the islands, 80% of *outside‐colony* locations were “open shrubland,” 60% of *near‐colony* locations were “tussock grassland–fernland” and 80% of *colony* locations were “succulent herbfield” (Figure 3). We therefore omitted habitat structure from our models.

### 3.1 Family‐level community structure

Across the sampling events, spider activity‐density was greatest on Whale Bone Point (11.8 ± 4.64 spiders captured island‐wide per trapping day) and lowest on Wedge Island (4.06 ± 1.65 spiders captured island wide per trapping day), with the differences between islands statistically significant (ANOVA, *F*_4,20_ = 4.51, *p* = .01) (Figure 3). There was no effect of sampling event (LRT, G_4_ = 2.05, *p* = .73) or colony status (LRT, G_2_ = 2.13, *p* = .34) on activity‐density. Visual examination of the data indicated that there may be an interaction between island and colony status. On Maatsuyker Island, spider activity‐density was highest in the *outside*‐*colony* location compared to the *colony* location with 5.60 ± 2.01 and 2.66 ± 1.65 spiders captured at each location each sampling event, respectively (Tukey's *t* = 2.81, *p* = .04). Across the other islands, spider activity‐density was greater at *colony* locations than at *outside*‐*colony* locations, with an average of 3.16 ± 2.43 SD and 1.60 ± 1.05 SD spiders captured per trap day each sampling event in each location, respectively (Tukey's *t* = −2.54, *p* = .04). Adding island into the model as a fix effect interacting with colony status confirmed the significance of this interaction (LRT, G_8_ = 27.12, *p* < .001) (Figure 3).

Rarefied family richness did not differ between sampling events (LRT, G_4_ = 4.80, *p* = .31), but did differ with colony status (LRT, G_2_ = 6.88, *p* = .03). Richness was greater at *outside*‐*colony* locations (1.79 ± 0.14) than *colony* locations (1.68 ± 0.18) (Tukey's, z = 2.39, *p* = .04). *Near*‐c*olony* locations did not differ significantly from the other two locations.

**FIGURE 4:**
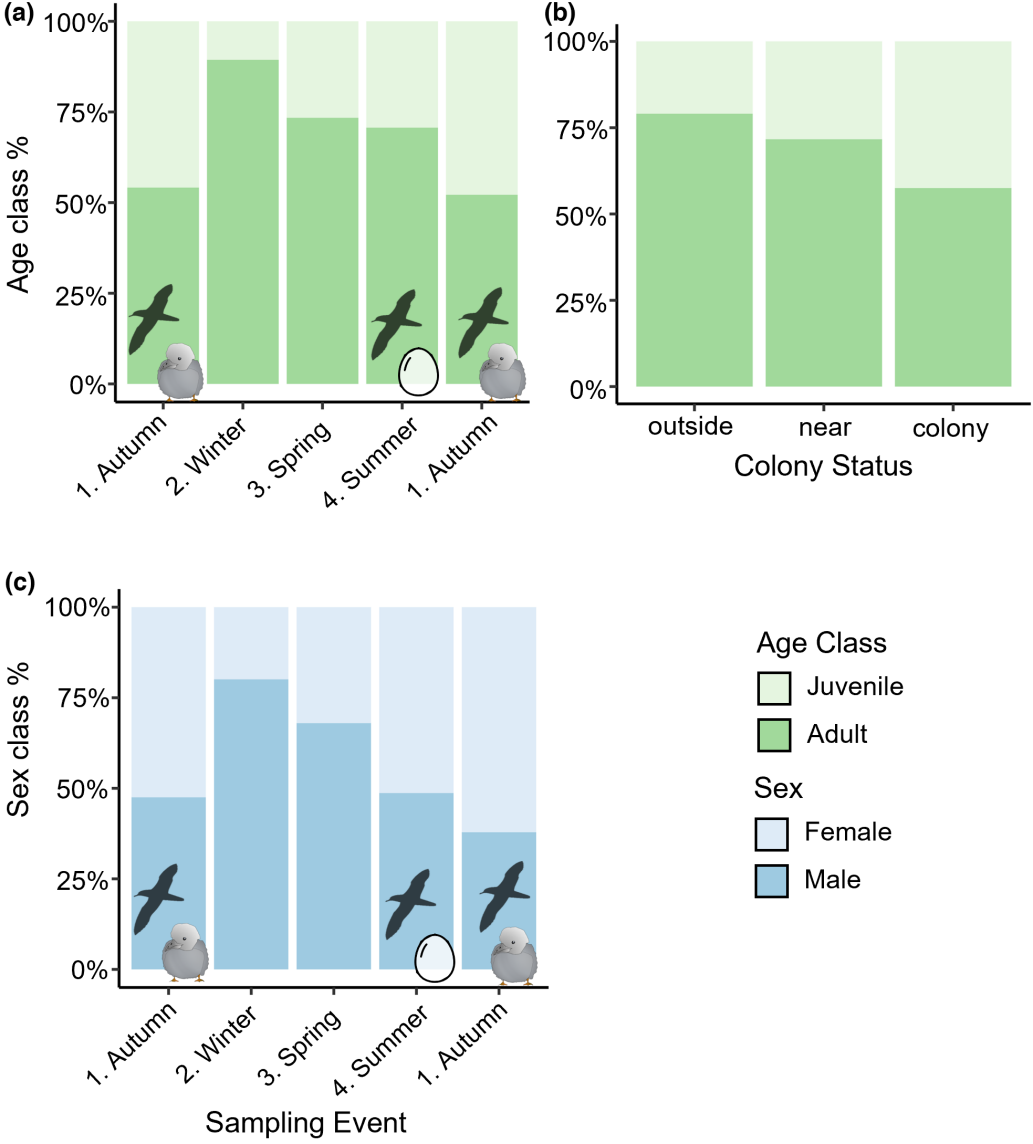
Proportion of adult to immature spiders caught across (a) sampling events and (b) colony status location. (c) Proportion of adult male to female spiders caught across sampling events. Seabird symbols indicate the stage of the short‐tailed shearwater breeding cycle at each sampling event.

Age class structure varied with sampling vent (LRT, G_4_ = 36.48, *p* < .001) and to a lesser extent colony status (LRT, G_2_ = 4.96, *p* = .08) (Figure 4). A greater proportion of spiders captured at *outside*‐*colony* locations were adults than in *colony* locations (Figure 4), although the difference was not statistically significant (Tukey's, z = 2.06, *p* = .10). The proportion of adult to immature spiders captured was most similar during the two autumn sampling events, with the proportion of adults captured the highest in winter (Figure 4). The proportion of males to females was also most affected by sampling event (LRT, G_4_ = 52.08, *p* < .001) with the fewest females captured proportionally to males in winter and spring (Figure 4). The winter sampling event was dominated by Linyphiidae, which comprised 42.5% of the adult spiders captured, of which 80.5% were male.

### 3.2 Family‐level community composition

**FIGURE 5:**
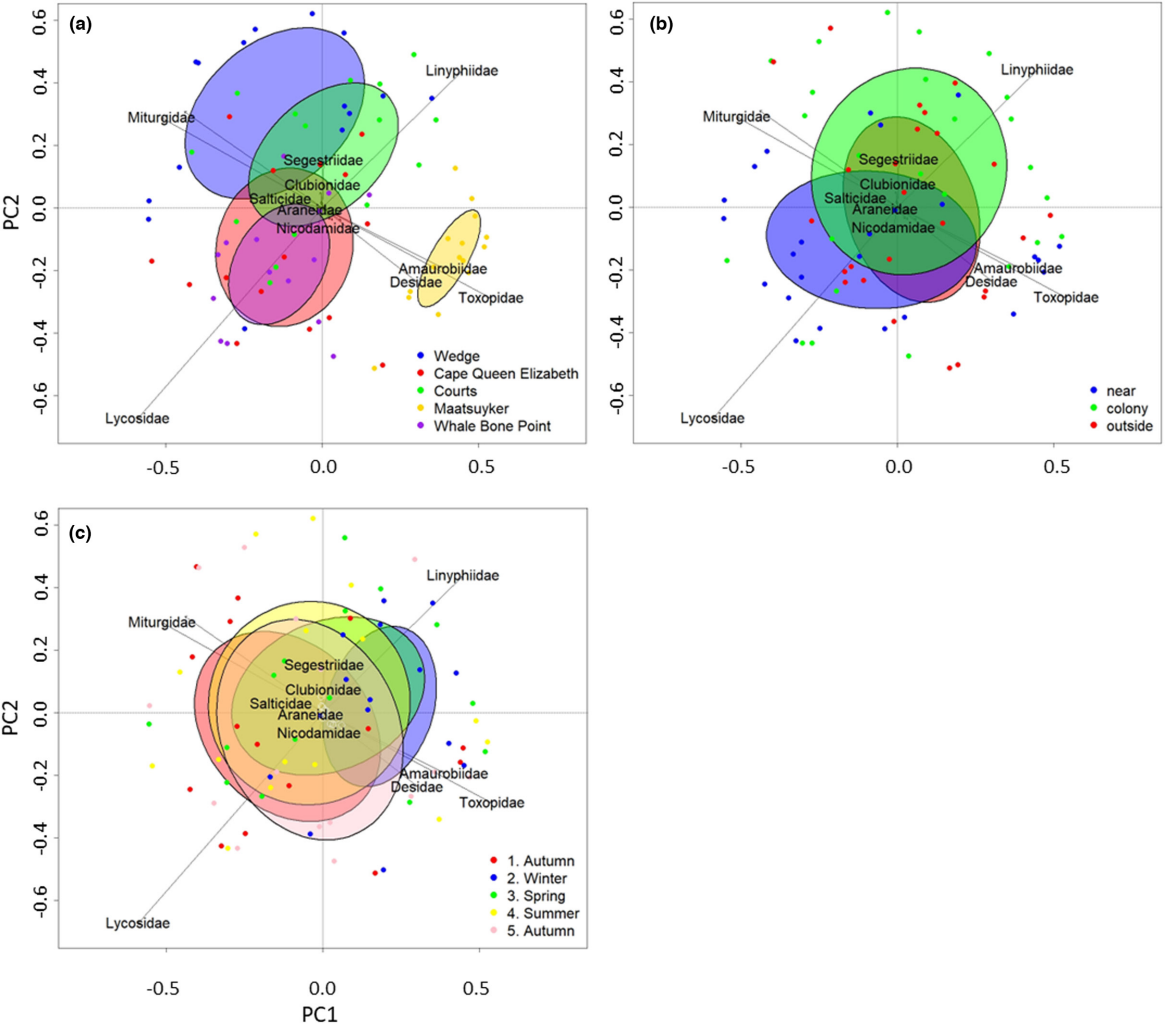
Results from principal component analysis (PCA) on the relationships between (a) island, (b) colony status, and (c) sampling event and spider family‐level community composition. PCAs were performed using scaled standardized spider activity‐density values (average number of spiders from each family caught at each colony status locations at each sampling event/number of trapping days) and a Hellinger distance matrix. Arrows represent spider families and points represent pitfall traps from the same colony status locations on each island and each sampling event. Ellipses represent one standard deviation from the centroid of each variable level.

Ground‐active spider family community composition was most distinctive between islands, while there was little difference between sampling events or colony statuses (Figure 5). PERMANOVAs confirmed these results, with the difference between groups more significant for island than by sampling event or colony status, though the latter were still significant (island: *F*_4,74_ = 6.79, *p* < .001, colony status: *F*_2,74_ = 1.82, *p* = .03, sampling event: *F*_4,74_ = 1.53, *p* = .03). Pairwise comparisons for all levels of each variable also supported the results from the PCAs (Supplementary S4). Maatsuyker island had the most distinctive spider family community composition (adjusted *p* < .001). Similarity was greatest between the two Bruny Island peninsular sites, Cape Queen Elizabeth and Whale Bone Point (adjusted *p* = .39). There was also similarity between Courts and Cape Queen Elizabeth (adjusted *p* = .190) and Courts and Wedge Island (adjusted *p* = .06). There was no difference in spider family community composition between the different colony statuses, and only a difference between sampling events 1 and 2 was identified (adjusted *p* = .003) (Figure 5).

## 4 DISCUSSION

### 4.1 Seabird colony presence and habitat structure

Short‐tail shearwater colonies are a common feature of each of the islands in this study. Excluding Maatsuyker Island, we found greater spider activity‐densities at *colony* locations compared to *outside*‐*colony* locations, and family‐level richness was generally greater at *outside*‐*colony* locations than *colony* locations. We also found the expected strong correlation between habitat structure and colony status. The trampling, nest building, burrowing, and high levels of nutrient input associated with seabird colonies can reduce habitat complexity, exposing bare ground and reducing plant species richness (Ellis, 2005).

Links between seabird presence and spider activity‐densities have been investigated in several other studies, with varying conclusions. In the Gulf of California, islands with seabirds supported greater spider numbers (Polis & Hurd, 1995), and within an island, spider densities were eight times higher inside than outside seabird colonies (Polis & Hurd, 1996). Contrastingly, in northern New Zealand, spider activity‐density was similar on islands with and without seabirds (Towns et al., 2009). In the Stockholm archipelago, different spider groups responded differently to seabird presence. Web‐spiders showed no difference in densities between islands with and without seabirds, while Lycosidae were lower with seabird presence (Kolb et al., 2010). The variable responses of spiders to seabird presence likely reflects the balance between increased productivity associated with the addition of nutrients from seabird guano and the disturbance inflicted by seabird burrowing and trampling. Spiders from *colony* locations on each of the islands in this study utilize seabird‐derived marine nutrient subsidies more than those found in *outside* or *near*‐*colony* locations (Pascoe et al., 2022). Spiders were similarly found to be utilizing more seabird‐derived food sources on active compared to abandoned cormorant islands in the Stockholm Archipelago (Kolb et al., 2010). The greater availability of seabird‐derived nutrient subsidies at *colony* locations may explain why more spiders were found at *colony* or *near*‐*colony* locations on all islands apart from Maatsuyker Island, where other factors, such as disturbance levels and habitat structure, may be having a greater effect.

Habitat structure is known to be a key determinant of invertebrate communities (Jonsson et al., 2009). Of all the sites, bare ground was most prevalent at the Maatsuyker Island *colony* location. The interior of Maatsuyker Island is covered in a dense canopied *Leptospermum scoparium* forest. Shearwaters nesting in this region of the island must walk through the forest each morning and evening to reach and return from areas suitable for take‐off and landing. This high physical disturbance from hundreds of thousands of birds is likely greater than at other island colony locations, creating less ground cover. This may drive the lower spider activity‐densities recorded. The *near*‐*colony* location on Courts Island and the *outside‐colony* location on Maatsuyker Island were the only two locations to have higher spider activity‐densities than their respective *colony* locations. At both locations, pitfall traps were within open shrubland, however they were located at ecotones (Maatsuyker Island—cleared lawn; and Courts Island—succulent vegetation), where greater habitat complexity is available. Ecotones have been found to support high spider densities and family‐level richness in other regions. For example, in Panama, spider web density was found to be greater at woodland edges (Lubin, 1978) and, in agricultural areas of western and central Germany, spider family richness and activity‐density were greater at the ecotones provided by the edges of fields (Clough et al., 2005).

### 4.2 Seasonality

We did not detect seasonality in spider activity‐density levels or family‐level richness associated with seabird seasonality. We anticipated potential seasonal change in spider communities in response to pulsed nutrient subsidies and disturbance levels associated with seasonality in colony attendance by the short‐tailed shearwaters. Pulsed disturbance was found to negatively affect spider densities in a simulated grazing experiment in Texan marshes, likely associated with a loss of shelter and prey reduction (Armitage et al., 2013). In seabird colonies, spider communities may be more adepted to this pulse style of disturbance as it occurs with such regularity, with vegetation characterized by the colonies' presence, rather than being seasonally affected by seabird disturbance. On arid islands in the Gulf of California, pulsed nutrient availability, dictated by a combination of marine subsidies and rainfall, which limits primary productivity in the region, influenced spider abundances (Anderson et al., 2008; Anderson & Polis, 2004). On the Tasmanian islands examined in this study, spiders in the colonies benefit from a food web supported by a seabird‐derived nutrient legacy year‐round (Pascoe et al., 2022). This may be sufficient to support the spider communities inside colonies year‐round. The reach of the seabird nutrient subsidies is also relatively limited on these islands (Pascoe et al., 2022), meaning that the absence of seabirds over winter may be expected to have little effect on *near* or *outside*‐*colony* spider communities.

Seasonal weather patterns also drive variability in spider communities. In Panama, web‐building spider activity‐density was lower in the dry season, when conditions likely reduced the availability of their prey, compared to the wet season (Lubin, 1978). In Mexican tropical mountain cloud forests, strong seasonal variability in understory dwelling spider abundance, community structure, and diversity were detected; however, little seasonal variability was detected in ground spiders (Campuzano et al., 2020). In temperate forests of Greece, seasonal differences were found in ground spider species richness, but not in abundance (Zografou et al., 2017). Tasmania has a cool temperate climate with low seasonal variation in rainfall and temperature. With all seasons likely to be sufficiently favorable for spiders and their prey, it is possible that seasonal variation in activity‐densities due to weather may not occur. Seasonality in spider family and species level richness however has been found for spiders in coastal heathlands in north‐eastern Tasmania, with more families sampled during summer months (Churchill & Arthur, 1999). While we did not detect seasonality in family‐level richness, it is possible that at a higher‐taxonomic resolution than family, seasonal differences would be detected (Churchill & Arthur, 1999; Driessen & Kirkpatrick, 2019).

We did detect seasonal differences in spider age and sex class structure, and a difference in spider family‐level community composition between the winter and other sampling events. This may reflect spider phenology and life cycle, rather than a response to seabird or weather seasonality. Spiders can be categorized into a range of life type cycles based on what stage they overwinter in, when reproduction occurs, and when adults and eggs occur (Schaefer, 1977). The dominant families found across these study islands, Linyphiidae and Lycosidae, are found in almost all terrestrial ecosystems (World Spider Catalog, 2022), and consequentially they have evolved life cycles that can adapt to a huge range of environmental conditions (Shah & Buhroo, 2022). More detailed phenological studies are required in these regions to make conclusions about the dominant life cycles of these taxa. In general, across the study islands, more males were caught during winter and spring, and the proportion of adult to immature individuals was also greater in winter and spring, extending into the summer sampling event. The family composition of the winter sampling event was characterized by a greater proportion of Linyphiidae. Many Linyphiidae species reproduce during winter and during mating males often move around in search of females (Marc et al., 1999), potentially explaining the high numbers of adult male Linyphiidae captured during the winter sampling event.

### 4.3 Interisland variability

Spider activity‐densities and family‐level community composition differed between the islands. Whale Bone Point had the highest spider‐activity density while Wedge Island had the lowest. Maatsuyker Island had the most distinctive family community composition, driven by a greater prevalence of spiders from the Toxopidae, Amaurobiidae, and Desidae families than the other islands. The composition of families was similar at Whale Bone Point and Cape Queen Elizabeth, and Courts and Wedge islands also displayed some similarity in their family‐level community composition, but to a lesser extent. These similarities and differences are likely driven by the characteristics of each island such as size, soil chemistry, vegetation community, as well as their isolation from other large land masses.

The theory of island biogeography posits that the composition of a given biological community is determined by immigration, extinction, and speciation, which are all affected by the size and degree of isolation (distance to mainland) of an island (MacArthur & Wilson, 1967). We did not detect clear patterns between island size and activity‐density or family‐level spider richness. Family‐level richness did not differ between the islands, possibly a product of the coarse, family‐level higher taxa surrogacy used in this study. Activity‐density was highest on Whale Bone Point, which is situated on a peninsular of the larger Bruny Island, however Cape Queen Elizabeth, also a Bruny Island peninsular site, was not consistently as high. The differences in activity‐density levels between the islands may be explained by differing levels of predation and competition experienced by spiders on each island. For example, across 106 islands in the Bahamas, a strong positive relationship was found between spider abundance and island area, except for islands also inhabited by lizards, where the effect of area was much weaker with lizards predating on the spiders (Toft & Schoener, 1983). Further research is required to understand the level of predation faced by spiders on each island, and how this may affect spider communities.

Island isolation may explain some of the differences and similarities between the communities. Island isolation can influence community composition by limiting dispersal. Maatsuyker Island is the most isolated among the study islands and had the most distinctive community. Whale Bone Point and Cape Queen Elizabeth, connected by land (26 km apart), had the most similar spider communities. Despite Courts Island and Whale Bone Point being the closest sites (9 km apart), Courts Island had a more similar family community composition to the other near‐shore island, Wedge Island (60 km away). While spiders are generally not highly mobile, they disperse in a range of ways. Some spiders use buoyant threads to cover hundreds of kilometers by “ballooning” on the wind (Bell et al., 2005; Decae, 1987). Spiders can also disperse via vectors such as seabirds or ships (Decae, 1987). Eight families were present on all five islands: Clubionidae, Desidae, Gnaphosidae, Linyphiidae, Lycosidae, Miturgidae, Theriididae, and Toxopidae. Linyphiidae, Lycosidae, and Theridiidae have previously been identified as being good dispersers (Blandenier, 2009; Richter, 1970), while Clubionidae, Gnaphosidae, and Miturgidae have been found to be less mobile (Carvalho & Cardoso, 2014). Generalizations at the family‐level about dispersal ability may be misleading though as species from within a family can differ in their dispersal capabilities (Juračka et al., 2019). The lack of similarity between close sites separated by water may suggest that dispersion over water or air is not a common occurrence in this area, potentially with spider families on each island representing what was there when each land mass became separated from the Tasmanian mainland, as the maximum postglacial marine transgression was attained around 6000 years ago (Dixon, 1996).

### 4.4 Sampling limitations

In this study, we aimed to provide an efficient and reproducible approach to assessing ground‐active spider communities across these islands. It was not our objective to obtain a comprehensive inventory of all spiders found on each island. We were not aiming to survey all habitat types. Our design enabled us to effectively compare spider communities between islands, intraisland locations, and sampling events to investigate the effects of colony status and seabird seasonality on ground‐active spider communities. We acknowledge pitfall trap design (shape, dimension, material, preservation substrate) can bias captures, and that they can bias adult males and larger taxa (as they are more active) (Hancock & Legg, 2012; Work et al., 2002). However, by using standardized sampling regimes and pitfall trap design between islands, locations, and sampling events, we were able to make reliable comparisons between these variables.

## 5 CONCLUSION

Here, we present the first insights into ground‐active spider communities on Tasmanian islands. We identified 26 families of ground‐active spiders across five Tasmanian islands where in most cases no previous records of spiders had been recorded. Despite limited taxonomic knowledge for spiders in this region, family‐level identification still provided ecological insights into spider community composition and structure and how each relates to the presence of seabird colonies and seasonality in this region. Our results provide some of the first insight into the spatial and temporal influences seabirds have on spider communities at the intraisland scale. We found that spider activity‐density was generally greater inside seabird colonies than outside, potentially reflecting the greater nutrient availability inside colonies. The exception was on Maatsuyker Island, where inside colony disturbance from seabirds was particularly high. Contrastingly, family‐level richness was generally higher outside seabird colonies, likely due to the greater habitat complexity found away from the disturbance of seabirds. We showed that for these islands, seasonality in seabird colony attendance did not affect activity‐densities, but there were some seasonal changes in age class and sex structure, potentially associated with the phenology and life cycles of the spiders themselves.

Spiders and seabirds are both critical components of many island ecosystems, yet the former are widely understudied, limiting our ability to fully understand island ecosystem functioning. These results provide additional insights into the relationships between seabirds and spiders and are also an important addition to the depauperate knowledge of spiders in this region, providing baseline occurrence data for future change to be assessed against.

## AUTHOR CONTRIBUTIONS

**Penelope Pascoe:** Conceptualization (equal); data curation (equal); formal analysis (equal); funding acquisition (equal); investigation (equal); methodology (equal); project administration (equal); writing – original draft (lead); writing – review and editing (equal). **Melissa Houghton:** Conceptualization (equal); investigation (equal); methodology (equal); resources (equal); validation (equal); writing – original draft (supporting); writing – review and editing (equal). **Holly P. Jones:** Conceptualization (equal); funding acquisition (equal); supervision (equal); writing – original draft (supporting); writing – review and editing (equal). **Christine Weldrick:** Conceptualization (equal); methodology (equal); project administration (equal); supervision (equal); writing – original draft (supporting); writing – review and editing (equal). **Rowan Trebilco:** Conceptualization (equal); formal analysis (supporting); methodology (equal); supervision (equal); visualization (equal); writing – original draft (supporting); writing – review and editing (equal). **Justine Shaw:** Conceptualization (equal); methodology (equal); supervision (equal); visualization (equal); writing – original draft (supporting); writing – review and editing (equal).

## Supporting information


Appendix S1.

Appendix S2.

Appendix S3.

Appendix S4.

## Data Availability

The data and code that support the findings of this study are available at: https://doi.org/10.25959/CSCA‐B214.
