# Supplementary material for: The effect of seabird presence and seasonality on ground‐active spider communities across temperate islands
Source: Ecol Evol. 2022 Dec 3;12(12):e9570. doi: 10.1002/ece3.9570 (PMC9719043; doi:10.1002/ece3.9570)

## Supplementary material 1 - Pascoe P. P., Houghton M., Jones H. P, Weldrick C., Trebilco R & Shaw, J. D. The effect of seabird presence and seasonality on ground-active spider communities across temperate islands

Characteristic genital structures used for assigning spiders to adult (including both sexually mature individuals with fully developed genital structures and subadult individuals with pronounced but not yet fully developed genitals), and immature (small individuals with no recognisable or very underdeveloped genital structures) age classes. A) adult female Lycosidae epigyne, B) immature female Lycosidae epigyne, C) adult male Lycosidae palp and D) immature male Lycosidae palp. Photographs: M Houghton


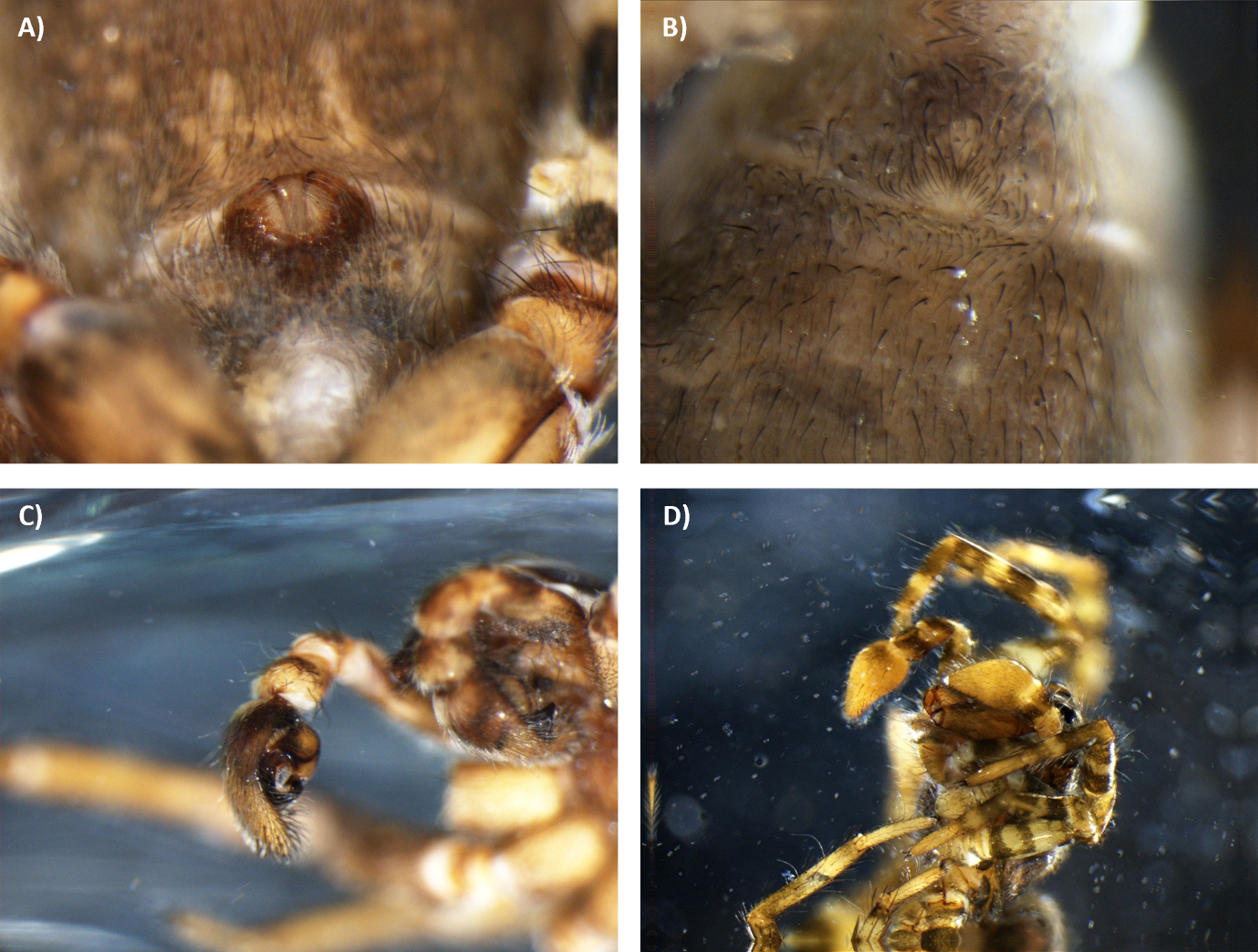

Supplement: Supplementary file 1 — Appendix S1. [file ECE3-12-e9570-s002.docx]
