# Supplementary material for: The effect of seabird presence and seasonality on ground‐active spider communities across temperate islands
Source: Ecol Evol. 2022 Dec 3;12(12):e9570. doi: 10.1002/ece3.9570 (PMC9719043; doi:10.1002/ece3.9570)

## Supplementary material 2 - Pascoe P. P., Houghton M., Jones H. P, Weldrick C., Trebilco R & Shaw, J. D. The effect of seabird presence and seasonality on ground-active spider communities across temperate islands

Results from non-metric multi-dimensional Scaling (NMDS) on the relationships between A) island, B) colony status and C) sampling event and spider community family composition. NMDS were performed using scaled standardised spider activity-density values (average number of spiders for each family caught at each colony status locations at each sampling event / number of trapping days) Ellipses represent one standard deviation from the centroid of each variable level.


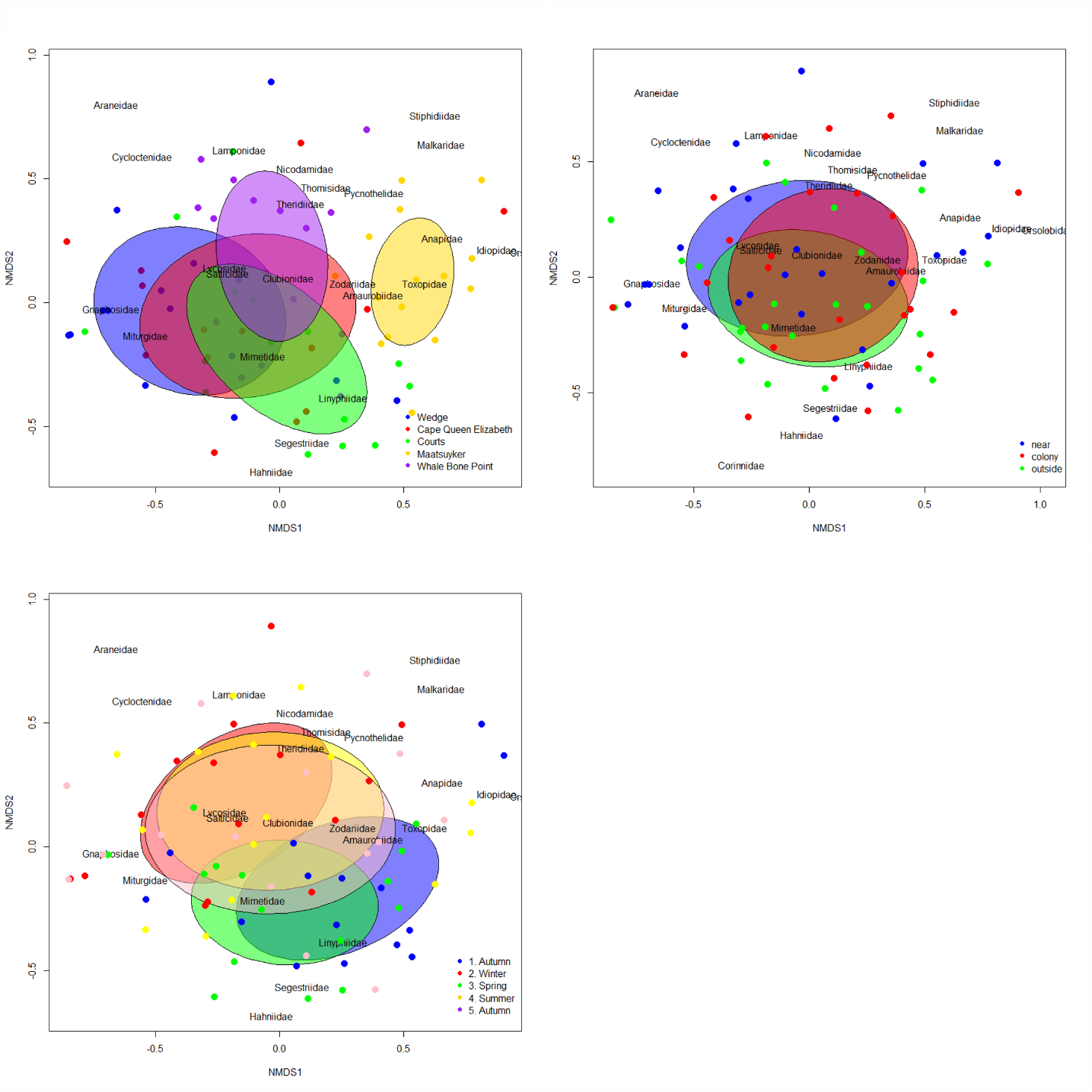

Supplement: Supplementary file 2 — Appendix S2. [file ECE3-12-e9570-s003.docx]
