# Supplementary material for: The effect of seabird presence and seasonality on ground‐active spider communities across temperate islands
Source: Ecol Evol. 2022 Dec 3;12(12):e9570. doi: 10.1002/ece3.9570 (PMC9719043; doi:10.1002/ece3.9570)
Supplement: Supplementary file 4 — Appendix S4. [file ECE3-12-e9570-s004.docx]

## Supplementary material 4 - Pascoe P. P., Houghton M., Jones H. P, Weldrick C., Trebilco R & Shaw, J. D. The effect of seabird presence and seasonality on ground-active spider communities across temperate islands

Pairwise comparisons for all levels of each variable (island, colony status & sampling event). Significant differences (P adjusted < 0.05) are highlighted in bold

| Pairwise comparisons | Df | Sums Of Sqs | F.Model | R2 | P value | P adjusted |
| --- | --- | --- | --- | --- | --- | --- |
| Islands | | | | | | |
| Cape Queen Elizabeth vs Whale Bone Point | 1 | 0.79 | 1.85 | 0.06 | 0.039 | 0.391 |
| Cape Queen Elizabeth vs Courts | 1 | 1.14 | 2.46 | 0.08 | 0.019 | 0.190 |
| Courts vs Wedge | 1 | 1.73 | 3.42 | 0.11 | 0.006 | 0.065 |
| Cape Queen Elizabeth vs Wedge | 1 | 2.07 | 3.96 | 0.12 | 0.000 | **0.004** |
| Courts vs Whale Bone Point | 1 | 2.06 | 5.06 | 0.15 | 0.000 | **0.002** |
| Cape Queen Elizabeth vs Maatsuyker | 1 | 3.82 | 9.90 | 0.26 | 0.000 | **0.001** |
| Courts vs Maatsuyker | 1 | 3.96 | 10.76 | 0.28 | 0.000 | **0.001** |
| Maatsuyker vs Wedge | 1 | 5.41 | 12.60 | 0.31 | 0.000 | **0.001** |
| Maatsuyker vs Whale Bone Point | 1 | 4.91 | 14.86 | 0.35 | 0.000 | **0.001** |
| Wedge vs Whale Bone Point | 1 | 3.09 | 6.60 | 0.19 | 0.000 | **0.001** |
| Colony Status | | | | | | |
| close vs no | 1 | 0.91 | 1.66 | 0.03 | 0.094 | 0.283 |
| colony vs no | 1 | 0.94 | 1.73 | 0.03 | 0.079 | 0.238 |
| close vs colony | 1 | 1.14 | 2.04 | 0.04 | 0.052 | 0.156 |
| Sampling events | | | | | | |
| 1 vs 4 | 1 | 0.47 | 0.85 | 0.03 | 0.546 | 1.000 |
| 1 vs 5 | 1 | 0.35 | 0.61 | 0.02 | 0.774 | 1.000 |
| 2 vs 3 | 1 | 0.75 | 1.49 | 0.05 | 0.140 | 1.000 |
| 3 vs 4 | 1 | 0.51 | 0.91 | 0.03 | 0.490 | 1.000 |
| 3 vs 5 | 1 | 0.48 | 0.85 | 0.03 | 0.553 | 1.000 |
| 4 vs 5 | 1 | 0.26 | 0.44 | 0.02 | 0.940 | 1.000 |
| 2 vs 5 | 1 | 0.99 | 1.80 | 0.06 | 0.069 | 0.689 |
| 1 vs 3 | 1 | 1.26 | 2.38 | 0.08 | 0.021 | 0.214 |
| 2 vs 4 | 1 | 1.27 | 2.36 | 0.08 | 0.012 | 0.117 |
| 1 vs 2 | 1 | 2.03 | 3.98 | 0.12 | 0.000 | **0.003** |
